# How Covid-19 changed emergency department access: observational study comparison of patient stage of the day access in the psychiatric emergency department over three years

**DOI:** 10.1192/j.eurpsy.2022.235

**Published:** 2022-09-01

**Authors:** R. Santini, T. Barlattani, T. Jannini, A. Mariano, F. Bianchi, C. Niolu, A. Siracusano

**Affiliations:** 1 University of Rome Tor Vergata, Department Of System Medicine, Rome, Italy; 2 University of L’Aquila, Psychiatry, L’Aquila, Italy; 3 University of Rome Tor Vergata, Department Of Systems Medicine, Chair Of Psychiatry, Rome, Italy

**Keywords:** accesses, emergency room, stage of the day, Covid-19

## Abstract

**Introduction:**

A few studies have analyzed the impact of COVID-19 pandemic on psychiatric Emergency Department (ED) accesses. The pandemic may indeed have influenced the phase of day accesses for patients with psychiatric disorders.

**Objectives:**

Aim of this cross-sectional study is to analyze how COVID-19 weighed on psychiatric patients daily accesses over the course of three years.

**Methods:**

Data on 219 patients were retrospectively collected from the ED in the Policlinico Tor Vergata, Rome. According to the stage of the day, accesses were divided into 4 groups: between 00:00 and 6:00; between 6:00 a.m. and 12:00 a.m.; between 12:00 a.m. and 18:00 p.m.; between 18:00 p.m. and 00:00 p.m.

**Results:**

Performing a regression analysis, a relation was found between psychiatric symptoms, stage of the day admission and year. In 2019 the admissions seem to be homogeneously distributed, however during 2021 and 2020 the admissions rates have a delayed evening trend.

**Conclusions:**

Despite the low number of accesses considered, the Covid-19 pandemic appears to exert an effect that still lasts in terms of both accesses and worsening or new onset of psychiatric symptoms. Measures taken to prevent the spread of infections may have affected access in the ED of patients in various ways. However, the trend of increasing evening accesses could be related to a saturation of territorial psychiatric services that work mainly until the afternoon. Thus, an enhancement of territorial psychiatric services seems highly necessary to cope with what could be an increase in psychopathology in patients without previous diagnosis.

**Disclosure:**

No significant relationships.

